# Palliative care and public health: an asymmetrical relationship?

**DOI:** 10.1177/1178224218819745

**Published:** 2019-02-20

**Authors:** Sandy Whitelaw, David Clark

**Affiliations:** School of Interdisciplinary Studies, University of Glasgow, Dumfries, UK; School of Interdisciplinary Studies, University of Glasgow, Dumfries, UK

**Keywords:** end of life, interdisciplinarity, palliative care, public health

## Abstract

Interest in the potential for public health and palliative care to work together is now widely established. Based on a mapping review of existing literature, we describe for the first time the ways in which public health has entered palliative care policy and practice and how this has been specifically articulated. We then go on to pursue analytical and critical lines of enquiry that are largely absent from the existing literature. We do this in three ways: (i) by considering why the link between public health and palliative care has become so ubiquitous within palliative care policy; (ii) by establishing how this has been constructed; and (iii) by exploring public health as a ‘reference discipline’ from which its ‘secondary deployment’ can become embedded *inside* another disciplinary field. From this, we develop a range of critical perspectives on the relationship between public health and palliative care by scrutinising its claims of utility and effectiveness and questioning the strength of the interdisciplinary interaction between the two disciplines. We see their relationship in a ‘cross disciplinary’ context which is still largely symbolic and tactical in nature. We conclude by considering the significance of these insights for policy and practice, with two possible scenarios. If the use of public health is essentially figurative and its resources are not unique, the particular and exclusive use of the term becomes insignificant. Progressive and effective policy and practice is possible, independent of any explicit public health label. If however public health is considered to have intrinsic and definable worth, we suggest that this currently asymmetrical association needs to be significantly developed with much higher levels of theoretical, practical and critical engagement between the two disciplines. Such work would result in more reflective and robust policy and practice.

## Introduction

The past 30 years have seen the emergence of a potentially productive interaction between the discipline of ‘public health’ and a range of health topic areas (such as obesity and drug misuse)^1,2^ and medical specialties (such as obstetrics and cardiology).^3,4^ One such expression has been in palliative care, where the recognition that death, dying, loss and care giving exist to some extent beyond the domains of individualistic therapeutic intervention, and therefore have something to gain from a closer association with public health.^5,6^ This relationship has to this point largely been couched in positive terms, wherein public health is seen as a compelling resource for shaping future directions in palliative care policy.^6^ This paper explores a range of more critical reflections with regard to making further improvements to the policy and practice that flows from this relationship.

We start by establishing an historical and definitional context, showing how public health has entered palliative care narratives and then more explicitly, clarifying the various forms of articulation that have been expressed in the existing literature. By exploring the deeper nature of the relationship and the drivers that have nurtured it, we then go on to pursue original and hitherto unconsidered analytical lines of enquiry that have the potential to impact on policy and practice. We do this in three ways: (i) by considering *why* the relationship with public health has become so ubiquitous within palliative care policy; (ii) by establishing *how* it has been constructed; and (iii) by understanding the implications of this particular engagement as a reflection of a wider theoretical trend, that sees public health as a ‘reference discipline’^7^ being used as a form of what we term, *secondary deployment* – wherein public health is embedded *inside* another disciplinary field.

Based on a series of expressed views that the association between public health and palliative care is still in its infancy and lacking in conceptual coherence,^8^ short of significant empirical evidence^9^ and practically bound by a series of ‘challenges’,^10^ we then go on to develop more novel ground in suggesting a range of critical perspectives on the relationship. Finally, we use this critical base to explore the fundamental directions this secondary deployment might take in the public health and palliative care context and consider how the relationship might be enhanced. We believe that, to date, the engagement has been somewhat rhetorical, rudimentary and overly optimistic, suggesting the need for more applied and critical forms of engagement between the two disciplines.

We conclude that this relationship might develop in two ways. If public health is essentially ambiguous, symbolic and not unique, the particular and exclusive use of the term becomes insignificant. In that context, progressive and effective policy and practice might be possible, independent of any explicit public health label. If however the public health term and its associated practice and knowledge base are considered to have intrinsic and specific worth, we suggest that there is a need to break through the superficiality and consensuality of current secondary deployment narratives and realistically accept the complex nature of the association and the possibility of divergent or even conflicting perspectives.

The material for this work was identified via a ‘mapping’ review^11^ that charts and categorises existing literature on a particular policy topic. We located a range of material across the period in which the public health-palliative care association developed, particularly reflecting an on-going intensification of interest over the past 10 years. The key pieces of literature are set out in Table 1. The majority of this material was of a theoretical and declaratory nature, with relatively little reporting on empirical practice. The aim of this paper is therefore to develop supportive and critical reflections of this ground.

**Table 1.** Core sources of review literature on the public health-palliative care association.

Paper	Type	Key aim
World Health Organization^12^	Policy statement	Reviews of the status of cancer care and pain relief and the production of recommendations and guidelines for improving the quality of life of cancer patients
Sepúlveda and colleagues^26^	Journal paper/policy position statement	Explores the development of palliative care through effective, low cost approaches
Kellehear^14^	Text book	Examines end-of-life care beyond palliative boundaries, describing a public health vision that involves whole communities adopting a compassionate approach to dying, death and loss
Stjernswärd and colleagues^6^	Journal paper/policy position statement	Describes a ‘Public Health Strategy’ (PHS) based on ‘four key pillars’: appropriate policies; adequate drug availability; education of policy makers, health care workers and the public; and implementation
Cohen and Deliens^15^	Chapter in edited text book	Describes the essential features of palliative care and public health and provides a sociological justification for adopting a public health approach to end-of-life issues
Clark^61^	Chapter in edited text book	Reviews the progress made in research into global palliative care development during the first decade of the new millennium, setting out the work that has been done by key non-governmental organisations to promote palliative care internationally
Gillies^41^	Briefing paper	Examines the rationale for applying a public health approach to palliative and end of life care, exploring where and how public health approaches could be applied to support local service planning and delivery
Sallnow and colleagues^5^	Journal paper	Reviews the evidence relating to the impact of a new public health approach to end-of-life care, specifically as this applies to efforts to strengthen community action
Abel and Kellehear^8^	Journal paper/policy position statement	Poses two key questions: how can we provide an equitable level of care for all people and how can we increase the range and quality of non-medical/nursing supportive care? Concludes that an important solution is adopting a public health approach to end-of-life care
Sallnow and colleagues^9^	Journal paper/policy position statement	Considers the research challenges related to examining new public health approaches to end-of-life care and how learning from more traditional or classic public health research can influence a future research agenda
De Lima and Pastrana^54^	Journal paper/policy position statement	Describes the history and evolution of palliative care (and associated systems failure) and goes on to propose a role for public health in palliative care integration in community settings
Archibald and colleagues^16^	Journal paper/policy position statement	Outlines a scoping review protocol that systematically maps and categorise the variety of activities and programmes that could be classified under the umbrella term ‘public health palliative care’
PHPCI^47^	Briefing paper	Offers a resource to enable further discussion on how public health approaches to palliative care can be developed to support people living with a life-limiting illness to maintain their quality of life
Dempers and Gott^17^	Journal paper	Describes the theoretical features of the public health approach to palliative care as articulated in the current research literature
Hazelwood and Patterson^10^	Journal paper	Describes the origins, rationale and work of Good Life, Good Death, Good Grief a national alliance of organisations and individuals working to promote open and supportive attitudes and behaviours relating to death, dying and bereavement; considers challenges and responses, achievements and learning
Abel and colleagues^21^	Journal paper/policy position statement	Describes how four essential elements within a public health model can work together to address quality and continuity of care as well as addressing the numerous barriers of access
Scottish Partnership for Palliative Care^44^	Briefing paper	Explores some of the different areas that can shape people’s experiences of death, dying and bereavement and what practical action might be taken to encourage and support open and supportive attitudes and behaviours

## A description of the evolution of the relationship

Over time, activists and commentators have suggested the need for palliative care to be understood as a public health issue.^6,23^ This originated in the 1980s when palliative care emerged as an innovative `field’ of intervention and first received recognition from the World Health Organization (WHO).^24^ Initially, this took the form of a focus on cancer pain relief,^25^ which included some public health values. The approach was predicated on three foundational measures, deemed low cost but capable of producing big effects: education (public and professionals); drug availability (changes to legislation and prescribing practices); and governmental policy (supporting the other measures).

In 1990, the WHO^12^ publication *Cancer Pain Relief and Palliative Care* maintained a focus on cancer, but engaged more widely with the question of palliative care by considering what could – and should – be done to comfort patients suffering from the painful symptoms associated with advanced malignant disease. In a context of recognising the scale and complexity of these problems, it marshalled arguments for palliative care based on the magnitude of unrelieved suffering borne by the majority of terminally ill patients. Although pain relief methods were emphasised, in the spirit of broadening the scope of approaches and in line with the holistic orientation of palliative care, other physical, psychological and spiritual needs were also included in the recommendations. The foundation measures were now applied to this broader set of goals. The work was a landmark in the history of palliative care, which had now been framed by the WHO as a global, public health issue and defined as:

The active total care of patients whose disease is not responsive to curative treatment. Control of pain, of other symptoms, and of psychological, social and spiritual problems, is paramount. The goal of palliative care is achievement of the best quality of life for patients and their families.^12^

Twelve years later in 2002, a new definition appeared from the WHO that further developed its public health character – particularly, introducing notions of prevention and early intervention:

Palliative care is an approach that improves the quality of life of patients and their families facing the problems associated with life-threatening illness, through the prevention and relief of suffering by means of early identification and impeccable assessment and treatment of pain and other problems, physical, psychosocial, and spiritual.^26^

Some welcomed this new language and the introduction of early identification of suffering as a pre-emptive measure.^12^ But the document itself expressed doubt over the depth and extent of the association, stating, ‘many countries have not yet considered palliative care as a public health problem and, therefore, do not include it in their health agenda’.^26^ These were large and complex issues, raising questions about the extent to which palliative care services were achieving their aims, either due to a lack of coverage or because the services did not have the capacity to deliver sophisticated multidisciplinary care.^26^

Adding further impetus to these processes, two special ‘public health’ issues of palliative care journals were published in 2007 and two edited collections on public health and palliative/end of life care appeared in 2012. Each sought to further articulate the association, albeit presenting two rather distinct discourses. Contributions to *The Journal of Pain and Symptom Management*^6^ described the aims of a public health approach as, ‘to protect and improve the health and quality of life of a community by translating new knowledge and skills into evidence-based, cost-effective interventions that will be available to everyone in the population who needs them’.

The policy narrative was affirmative and exhortatory, asserting that public health, ‘offers the best approach for translating new knowledge and skills into evidence-based, cost-effective interventions’.^6^ These strategies would be most effective if they involved the wider society through collective and social action. The approach therefore sought to ‘mainstream’ palliative care within national health care systems by involving wider stakeholders in the strategy. To this end, a fourth foundation measure was added: ‘implementation’.^6^

At the same time, the journal *Progress in Palliative Care*^27^ produced a special issue to ‘consider the practical, ethical, clinical and research issues arising from construing palliative care from a public health perspective’. Less normative and exhortatory than *The Journal of Pain and Symptom Management*, the emphasis here was more wide-ranging and reflective – looking to explore and raise questions rather than to declaim solutions. Published within a month of each other, these special issues each had a progressive orientation, pointing to both a range of topics deemed relevant to the linkage of public health and palliative care, as well as hinting at subtle and emergent tensions.

These points of difference can be seen more visibly in two edited volumes published in 2012 that set out a more detailed account of the association between public health and palliative care. The first^15^ expressed a concern that palliative care is still not well understood, needing both greater integration into health care systems and clear and measurable outcomes. It saw public health as a complement or counterpoint to the dimension of individual patient care, providing ‘the combination of sciences, skills and beliefs that is directed to the maintenance and improvement of health through collective or social action’.^15^

Focussed on the same topic, the second collection^28^ offered a different perspective on what foundation is best suited for palliative care. It began from a perspective that for much of life (including its ending), most people have little engagement with health care professionals and services. Rather, they encounter illness, loss and mortality predominantly as social experiences, shaped by culture, geography, beliefs, communities and relationships. Consequently, there is a belief that if palliative care is to build on its achievements and meet the challenges that remain, it must, ‘move beyond the bedside’ and embrace the challenge of community engagement by building on the ‘conceptual congruence between palliative care and public health’.^28^

In this edited collection it was noted that unlike the field of ‘health promotion’, with which it has much in common (e.g. both fields developed as a reaction against too much emphasis on disease, treatment and cure), palliative care had been slow to develop an international, structured global movement, the key concepts of which can be integrated into health systems.^29^ A key step in overcoming this was to occur in 2014 when the World Health Assembly^30^ passed a Resolution calling on all governments to integrate palliative care into their health care plans and policies and requesting the Director General of WHO to monitor global palliative care, evaluating progress made in collaboration with Member States and international partners.

These developments have been associated in turn with a range of sub-themes. These include moving palliative care beyond its original focus on cancer to include other diseases;^12^ the introduction of the notion of the ‘early intervention’ of palliative care in the illness trajectory;^31^ adopting ‘whole society’ and ‘compassionate communities’ orientations;^14^ stressing the principles of evidence-based and cost-effective interventions;^32^ and recognising the need for the integration of palliative care in wider health and social care systems.^15^ Additional sets of publications^33,34^ and most notably, a themed edition (‘Public health approaches to palliative care’) of *the Annals of Palliative Medicine* in 2018 contain further contributions to the debate. Most profoundly, Abel and colleagues^21^ re-assert the theoretical value of the association between public health and palliative care and re-affirm the core domains within it. In reflecting on the favourable political status of the public health-palliative care association, Marshall and Kortes-Miller^35^ report affirmatively on the 5^th^. International Public Health Palliative Care Conference. Finally, Horsfall^36^ and Librada Flores and colleagues^37^ describe specific project work. In their desire to ‘showcase’ this range of activity, Abel and colleagues^21^ suggesting the existence of a ‘tipping point’ in the implementation of public health led palliative care and offer ‘new essentials’ and ‘roadmaps’. It is a collection containing continued optimism about the association between public health and palliative care and the benefits that can accumulate. Thirty years on from the initial engagement, it seems that public health led palliative care is achieving full visibility as a global public health concern.

## Palliative care in the context of a ‘new’ public health and associated typologies

Beyond these generalised trends, some have attempted to define the nature of the public health-palliative care association with respect to two foundational bases. Primarily, this has been done in relation to the notion of a ‘new’ public health.^38^ Frenk^39^ has noted the proliferation of multiple and often unconnected expressions of a ‘new public health’ that have happened over many years and the ‘dangers’ of the addition of such an adjective. Some essential themes can however be detected within this essentially ‘post-Ottawa’ era that distinguish it from more ‘traditional’ public health, namely an acceptance of the comprehensive nature of health domains and determinants; particularly the significance of its social dimension; and the centrality of individual and collective enablement and advocacy in public health action in the context of community development for health.

The actual articulation has then been considered in relation to a series of comprehensive typologies.^17^ These tend to replicate the earliest attempts to model the generic field of ‘public health’ and ‘health promotion’,^40^ with a multi-faceted structure made up of broad dimensions like, ‘prevention’ (interventions aimed at avoiding contact with risk factors), ‘health education’ (efforts to change knowledge, attitudes and behaviour) and ‘health protection’ (legal or fiscal controls).

In review, these frameworks tend to reflect three broad ‘types’. First, a *practice*-driven approach is evident that treats public health as primarily a pluralistic concept, accommodating *varied* activities that are relevant to palliative care. Dempers and Gott^17^ term this a ‘World Health Organisation approach’ in that it acknowledges explicitly the ‘diverse’ WHO model cited above. At worst, these actions are simply a series of *ad hoc* parts, such as ‘appropriate policies’; ‘adequate drug availability’; ‘education of policy makers, health care workers, and the public’; and ‘implementation of palliative care services at all levels throughout the society’.^6^ At best, they are conscious of a need for what Gillies^41^ has termed a more systemic, comprehensive ordering and integration of these parts.

Second, a *methodology* driven orientation can be detected that draws on the traditional health metaphor ‘addressing contagion’^42^ as the most fitting conceptual resource. This approach envisions public health as a stepped *process*; typically starting with a classic pathologically oriented ‘epidemiology’ and ‘needs assessments’ of the scale and nature of ‘the problem’ before moving on to a progressive series of solutions such as developing an action plan, establishing planning mechanisms and developing practical components, with appropriate evaluation.^6^

Third, a more focussed ‘health promotion approach’ can be identified that emphasises various salutogenic notions, such as promoting community engagement, nurturing compassionate communities and adopting more progressive asset-based and social capital oriented interventions to palliative care.^22,43^

For some, the central focus of a public health led approach to palliative care is individuals in a clinical context – ‘a medical patient-caregiver approach’.^15^ Alternatively, a re-orientation model is proposed that acknowledges how care at times of loss is not simply a matter for formal services, but is ‘everyone’s responsibility’.^43^ Indeed, this latter position explicitly separates itself from clinical concerns. For example, the Scottish Partnership for Palliative Care^44^ states, ‘public health palliative care approaches *are not about*: therapeutic interventions with individual patients, group therapy, improving how a service delivers therapeutic interventions, creative or unusual ways of delivering therapeutic interventions’. Finally, some portray the interaction as existing across all of these domains: ‘organising the coordination and interaction between specialist palliative care, generalist palliative care, compassionate communities and the civic approach to end-of-life care – the *seamless* integration of these four components’.^21^

We now want to move beyond these largely positive and exhortative features in the literature to explore in more depth a series of features associated with the process of the emergence of the relationship between public health and palliative care that have not previously been given consideration. Rather than being simply natural and obvious, we see the association as socially constructed and in offering critical perspectives upon it, we seek to understand the basis of this construction. We therefore consider *why* the relationship has arisen, explore *how* it has been constructed; and ultimately locate it in the wider theoretical context of ‘reference disciplines’ and the notion of ‘secondary deployment’.

## Why the association has emerged

The literature reflects a strong belief that public health has inherent utility, possessing a range of valuable properties, such as being progressive in advancing prevention and early intervention approaches, possessing strong evidence-based underpinnings and adopting multiple and robust ‘ecological’ practices, particularly those that foster broad community participation.^45^

It should also be recognised that public health and palliative care each emerged as intervention fields around the same time in the 1980s. Their conceptual bases were shaped by common WHO values and both were associated with, but differentiated from, a medical model. Also, they both promoted early intervention and socially oriented approaches and were sympathetic to interdisciplinary working. As such, public health and palliative care may simply be seen as congruent with one another in some informed yet still ‘common sense’ manner.^27^

However, the ambiguity reflected in the definitions and typologies we have described suggests that the association is not simply ‘natural’ but is being actively constructed towards achieving particular ends. The existence of ‘policy symbols (as) political devices’^20^ that possess political capital has become a recognised feature within policy theory. Likewise, there is an acknowledgement that public health may possess a symbolic status in the form of ‘ritualistic’ policy-making^46^ involving an on-going production of policy documents. Palliative care policy has indeed been actively located in this discursive context, not least through its on-going commitment to the use of symbolic language^47^ and overt declarations of purpose.^48^

In this context, some see contemporary public health as a particularly favourable ‘signifier’, possessing ‘conceptual weight and influence’.^49^ Palliative care is also seen to have a relatively weak policy position, ‘poorly framed within evidence-based global policy-making’.^15^ In this context, in seeking greater recognition within public policy, the value of turning to supportive frames is therefore an option, with ‘public health’ as one possibility^15^ or indeed with ‘human rights’ as another.^50^ Protagonists in the field may therefore have concluded that as a ‘master’ frame,^51^ for palliative care, associations with public health are just as valuable in *symbolic* terms as they are functionally.

## How the association has been constructed

Turning to the matter of understanding *how* protagonists attempt to bolster the credibility of palliative care as a ‘public health issue’, we identify a series of specific mechanisms. The most prominent is the use of a ‘public health catastrophe’ narrative^52^ that acts as a device in both constructing significance and in stimulating policy development. For example, we see this elsewhere in relation to such phenomena as obesity^18^ and tobacco control.^53^

A series of constructed steps can therefore be observed here. To foreground an argument for palliative care, there is a tendency to emphasise the scale and severity of the issues it faces – explicitly aligning it with other significant issues such as HIV/AIDS, malaria, environmental pollution and cancer as being at least equal in severity and significance.^26^ These principles are then applied to palliative care, citing a host of problematic and dramatic dynamics such as global population growth and ageing, along with the magnitude of unrelieved suffering for which palliative care is proposed as the solution.^54^ Perhaps, the best specific example of this type of narrative can be seen in relation to *The Lancet Commission*^55^ and its notion of the existence of a ‘broad and deep … access abyss’ around palliative care and pain relief. This ultimately provides a basis for establishing an explicit link to palliative care being framed as ‘a *true* public health crisis’.^56^ Thereafter, the need to deploy a public health approach to address this scale and complexity is considered indisputable.^10^

## Reference disciplines and secondary deployment

A further aspect of our analysis centres on the fact that this type of policy narrative explicitly combines two distinct disciplines. The tendency for public health to be associated with other areas has a broader history, starting in the early 2000s with the emergence of the expression, ‘we need to adopt a public health approach to …’. We define this as a form of ‘secondary deployment’, a growing yet critically under-examined tendency wherein a remarkable range of issues is associated with public health, indicatively: drug misuse;1 obesity;^2^ HIV/AIDS;^57^ homelessness;^58^ climate change;^59^ violence against women^60^ and even bed bugs.^94^

These multiple examples suggest that many see public health as what has been termed a ‘reference discipline’.^7^ This is described as: ‘those disciplines X that provide foundational, methodological, or other inputs to another discipline/s Y such that the state of knowledge in Y is advanced through inputs provided by X’.^7^ In this context, public health is not just seen as *one* possible resource, but often *the only* one; for example, Ebbeling and colleagues^19^ suggesting that public health is ‘uniquely positioned’ to tackle the contemporary issue of childhood obesity. We sense a similar orientation has now developed in the palliative care field as it looks to public health as a key solution to the challenges it faces.

## Critical perspectives on the relationship

We believe that the association between public health and palliative care is not self-evident. Rather, it is constructed, relatively new and still developing.^16^ Some have therefore acknowledged the complexity of bringing together two multi-faceted fields whose concepts and boundaries are themselves highly debatable.^6^ Building on this, we suggest further lines of thinking.

A fundamental disjuncture in the relationship between public health and palliative care can be recognised. Palliative care can often remain insular and unsophisticated in its use of public health concepts.^61^ More profoundly, based on an almost complete absence of any specialist public health involvement in the material we have reviewed here, compared to other topic areas, public health seems relatively disinterested in palliative care as an issue of significance. For example, in our own country of Scotland, despite significant conceptual and practical exploration of the association within palliative care,^10,41,44,62^ a recent Scottish Government^63^ document setting out current national ‘public health priorities’ does not include any mention of end of life issues or palliative care. Put simply, the relationship can be considered asymmetrical, palliative care appearing to need public health more than the converse.

While many have called for a better ‘alignment’ of public health and palliative care,^5^ the essential direction of the relationship still appears underdeveloped, even muddled. Those within palliative care have foregrounded public health as the referent discipline, seeking to bring its values *into* another host discipline;^5–7^ for example suggesting, ‘(this) approach takes the principles of health promotion and *applies them to* addressing the morbidities and mortalities associated with death, dying and loss’.^6^ Others reverse this relationship, foregrounding palliative care as the predominant discipline expressed *within* public health, for example, seeing ‘opportunities for palliative care *in* public health’.^17^

The former suggests a relatively conservative form of engagement limited to care, while the latter hints at a more fundamental re-orientation of practice where the shape of palliative care is significantly altered. This has generated certain ambiguities and potential conceptual blurring,^64^ reflected in confusing *non sequiturs*; for example, ‘a public health approach to palliative care is a health promotion approach to end of life care’^47^ and ‘a public health approach to palliative care necessarily adopts the tenets of both palliative care and health promotion’.^6^ More profoundly, the concepts of ‘public health’ and ‘health promotion’ are often used interchangeably with little definitional foregrounding.

In this context, the expression of disciplinary ‘public health’ itself can be considered relatively rudimentary. By looking mainly for pragmatic solutions, we believe that palliative care has taken public health at face value as a formal ‘science’ rather than the quasi-religious ‘contemporary cult of humanity’ identified by Dew.^65^ It has also largely ignored or bypassed a host of complexities and tensions that are prominent within public health itself. Some doubt the possibility of public health having a single indisputable essence – what Hamlin^66^ calls an ‘eternal form’, favouring a position that sees public health as an amalgam of potentially divergent values – individualistic *and* collective activity; pathological *and* salutogenic orientations; whole population *and* targeted methods; authoritarian *and* negotiated approaches.^67^

The extent to which such theoretical possibilities actually reflect the range of actions that are undertaken has also been questioned - the tendency being for individualistic elements to be over-represented at the expense of those addressing social and structural features.^68^ Such established critical concerns are generally disregarded by palliative care activists, seen for example in the absence of any consideration that public health practice can in theory be ineffective,^69^ unethical,^70^ or even harmful.^71^

The bulk of the palliative care narrative in this context is instead derived from relatively narrow and consensual sources – the WHO, its 1986 *Ottawa Charter* and the notion of ‘three waves’ of public health that dominated public health discourse in the 1990s and early 2000s.^14^ Such perspectives overlook significant critiques of the excessive influence that the WHO has had in conceptualising public health, particularly the ‘western’ orientation of the *Ottawa Charter*.^72^ In limiting the source material to the 1990s and early 2000s, there is also little evidence of any effort to see public health in relation to two highly significant emergent domains. The first is the contextualisation of public health in relation to a ‘4th wave’ and the contemporary forces of late modernity, globalisation, consumption and environmental consequences that currently illuminate much social and political debate.^73^ The second is the emergence of ‘systemic’^74^ or ‘ecological’^75^ perspectives, concerned explicitly with complex policy implementation and involving the co-ordinated interaction of multiple strands of action or as a mechanism for multi-faceted policy development in public health.^76^

While the normative aspiration of ‘an integrated approach’ is very prominent in the public health-palliative care literature, there has been very little attention paid to its actual mechanisms, beyond aspirational generalities. Consider by contrast cognate areas such as obesity prevention,^77^ physical activity promotion^78^ and educational development^79^ that have actively and critically sought to articulate the nature of a public health oriented system in their particular domain.

The general and declaratory nature of this secondary deployment narrative also seems to come at the expense of significant reporting or evaluation of actual public health actions and practices.^6^ When this does occasionally happen, examples tend to be isolated and pertain to relatively low level and localised *ad hoc* public health activities and projects, for example, community interventions and societal education.^5^

In turn, rather contradictory stances are adopted on the robustness of the evidence base associated with such work. Commentators have expressed concerns about various aspects of this ground. Sallnow and colleagues^9^ are troubled about the basic infrastructure to support such work, critically concluding that ‘there is little evidence of a dynamic research agenda to measure and evaluate the proliferating new public health efforts around end of life care’. Archibald and colleagues^16^ focus on the appropriateness of the particular approaches that are used, stating ‘there is not widespread clarity about how these approaches can be undertaken in practice or how evidence can be gathered relating to the effectiveness of these approaches’. Hazelwood and Patterson^10^ point to the consequences of this, namely ‘a shortage of evidence specific to public health approaches to palliative care’. Abel and Kellehear conclude that ultimately ‘the public health movement in palliative care … its practices, rationales, and impact remain poorly understood’.

Yet many still feel able to talk affirmatively about the strength of what is known. For example, Sallnow and colleagues^9^ feel ‘evidence is emerging to support the theory and aspirations of new public health approaches’ and Abel and colleagues^21^ contend the field ‘has shown promising evidence of effectiveness’. These assertions exist ‘not withstanding’ the problems that Sallnow and colleagues^9^ recognise in relation to the limitations of the evidence base and appear to be based on a small number of generic public health reviews and *ad hoc* empirical case studies, rather than public health as a comprehensive and systematic phenomenon that needs to be evaluated systemically.^80^ We therefore suggest that the extent and robustness of the interaction between public health and palliative care is not as strong as many have implied.

A hierarchy of disciplinary connectedness sees ‘multi’, ‘inter’ and ‘trans’ disciplinarity as escalating levels of interaction.^81^ Here, the weakest expression is ‘cross-disciplinarity’, seen as simply ‘the viewing of one discipline from the perspective of another’.^81^ Based on the public health oriented palliative care literature having little direct engagement with disciplinary or practice-based public health, we locate the relationship in this category. It constitutes a relatively superficial, one-way borrowing that has so far, largely foregone the possibility of deeper mutual interaction.

## Discussion

Pragmatic perspectives would see public health as a significant resource for palliative care policy, based simply on its utility. However, recognising the symbolic basis of public health described here, and the potential it has at times to be a populist rhetorical ‘buzzword’,^82^ does draw this assumption into question. The symbolic basis of the relationship tends to foster a context in which non-specific and exhortative claims appear to be considered sufficient and not open to question.^82^

The literature we have reviewed is largely characterised by its absence of critique and by its consensuality, displaying few tensions. This might be seen as politically constructive, enabling ‘the transformation of individual intentions and actions into collective results and purpose … without [which], co-operation and compromise would be far more difficult, if not impossible’.^83^ However, seeing the multidimensional aspects of public health in blended, consensual terms^8^ tends to forego any healthy critical assessment and ignores the existing prominent critiques of public health ‘eclecticism’^84^ and characterisations of the discipline as an ‘interdisciplinary magpie … collecting … trinkets from other disciplines’.^85^ Ultimately the shallowness of engagement between public health and palliative care precludes deeper disciplinary interaction and meaningful exchange of ideas and potential conflicts that come with ‘multi’, ‘inter’ and ‘trans’ disciplinary work.^86^ Its symbolic ambiguity creates what Jones and Greene have termed, ‘an illusion of shared meaning’.^52^

We argue that beyond the simple notion that the promulgation of ‘big problem’ claims can gain status and prominence for a topic area and prompt ‘big actions’, a range of difficulties can be associated with this orientation when it comes to public health and palliative care. By striving for ‘problematic’ status, there is a risk of exaggerating the core scale of the problem.^87^ Some also point to the fallacy of simply transposing public health solutions from one area to another.^83^ Likewise, the propensity to over-estimate the claimed success of public health approaches in the referent area is acknowledged.^88^

A case can therefore be made that the public health narrative within palliative care seeks to inflate the scale of end-of-life problems, and to assume the solutions that worked in spheres such as HIV/AIDS, malaria, environmental pollution and cancer will be similarly effective in palliative care.

The attractiveness of utilising public health in a range of cognate areas might appear self-evident. Its progressive ethos and ecological practice have the *theoretical* potential to promote more sophisticated forms of policy and practice. We have shown how this has been expressed within palliative care, how the connection has been promulgated, recognising that the evidence base for theory and practice in this domain is growing.^9,10^ Yet, beyond this affirmative ground, we have highlighted three critical concerns.

First, drawing on the current capital that public health appears to possess, we suggest that its deployment in palliative care is as much symbolic as pragmatic. We believe that this could create an ‘empty’ context into which protagonists can project themselves without needing to operationalise their thoughts. In by-passing both the profound socially oriented roots of public health and the internal critiques *within* the discipline that are so crucial in grounding realistic practice, there is a risk of vague public health – palliative care articulations.

Second, the extent to which the value of public health has attained a ‘common sense’ status in cognate areas should be made problematic. The nature of such embeddedness means that deep assumptions become taken for granted and uncritically accepted. In attaining conceptual transparency and avoiding potentially fallacious conclusions, such assumptions need to be questioned.

Third, in locating the relationship between public health and palliative care in a ‘cross-disciplinary’ context, we show that the links between the two tend to be rudimentary: ‘boundaried’ rather than ‘permeable’. In these circumstances, we note that there are very few examples of genuine interdisciplinary interaction between public health specialists and those within palliative care^89^ with Abel and Kellehear^90^ recently re-enforcing this notion in relation to their recognition that the public health-based well-being models and methodologies for social support are weak within the UK Palliative Medicine Syllabus. We therefore encourage more nuanced and reflective engagement.

## Conclusion

This article has critically explored the cross-disciplinary association between public health and palliative care as a form of ‘secondary deployment’. It has suggested that the basis of this relationship is not as robust as has been implied. As such, this association could be progressed in two directions: developing stronger palliative care policy and practice independent of any specific `public health’ label; or deploying useful resources from the field of public health to advance progressive palliative care.

This would mean a genuine acceptance of the complex nature of the association^91^ and the possibility of divergent or even conflicting perspectives within public health. It would also involve some classification and accommodation, for example, between those who advocate for a public health approach to palliative care service development within the wider health and social care system, compared to those who see the public health approach through a much wider lens incorporating multi-faceted actions and perspectives, as well as community and lay viewpoints.^10^

Such work would actively look to promote what Tormey and colleagues^62^ term ‘interdisciplinary literacy’ and some progress in this respect is clearly starting to happen. For example, in relation to basic disciplinary engagement, Gillies^41^ reports on a process in Scotland that meaningfully brought together public health and palliative care specialists and began to explore the complexity of how public health and palliative care can relate to one another. This involves charting available public health approaches, identifying the core elements of a public health orientation and critically recognising associated challenges.

This approach has nurtured the development of a series of initiatives within Scotland from Hazelwood and Patterson,^10^ Gillies,^41^ The Scottish Government^92^ and the Scottish Partnership for Palliative Care^44^ that are particularly progressive in starting to offer a theoretically grounded, comprehensive, reflective and practical review of the possibilities of such a public health approach. In relation to the need to realistically address the complexity of the public health-palliative care relationship in a systematic way, Hazelwood and Patterson^10^ have also critically accepted the ‘challenge’ of the ‘breadth of this agenda’ and the need to manage the ‘multiple strands of public policy’ that a public health approach to palliative care inevitably opens up. There is a need to actively engage with this as a complex policy process - rather than a set of isolated actions. Henry and colleagues^91^ have demonstrated the value of this orientation in a critical policy analysis of public health-based approaches to improving access to palliative care for homeless populations.

But these are isolated examples and we believe more is required. Further critical review is needed in both theoretical^93^ and pragmatic^17^ terms, exploring and clarifying key domains. In foundational terms, there needs to be an open and honest setting out of the core nature of the constituent elements of ‘public health’ and ‘palliative care’, with an acceptance that there are contrasting and potentially conflicting readings of these disciplines and a recognition that public health is not implicitly constructive. A genuinely detailed and reflective exploration of the range of potential interactions between the disciplines is also required. This should be done at both theoretical and practice levels, with greater actual collaboration between public health and palliative care specialists. Finally, a more systematic orientation is needed, one that reflects on the ways these multiple interactions can be optimally co-ordinated. If these are undertaken with rigour, the engagement between the two fields may yet prove fruitful.
